# An examination of willingness to participate and willingness to pay for a universal school food program in the Canadian context

**DOI:** 10.1017/S1368980023002070

**Published:** 2023-12

**Authors:** Suvadra Datta Gupta, Punam Pahwa, Rachel Engler-Stringer

**Affiliations:** 1 Department of Community Health & Epidemiology, University of Saskatchewan, HSC E-wing 3214, 107 Wiggins Road, Saskatoon, Saskatchewan S7N 5E5, Canada; 2 Canadian Centre for Health and Safety in Agriculture (CCHSA), University of Saskatchewan, Saskatoon, Saskatchewan, Canada

**Keywords:** Willingness to participate, Willingness to pay, School food program, Contingency evaluation, Double hurdle model

## Abstract

**Objective:**

To examine parents’/caregivers’ willingness to participate and willingness to pay (WTP) for a cost-shared school food program (SFP) and its associated factors.

**Design:**

A quantitative survey design was used where WTP for a hypothetical SFP was elicited using a double-bounded dichotomous choice elicitation method. We used a double hurdle (logistic and truncated regression) model to examine WTP and positively or negatively associated factors.

**Setting:**

Saskatoon Public School Division elementary schools situated in high-, mid- or low-median-income neighbourhoods.

**Participants:**

Parents or caregivers of children attending grades 1 to grade 8 in the Saskatoon Public School Division elementary schools.

**Results:**

94 % respondents were willing to participate in a SFP while less than two-thirds of participants were willing to pay for such a program. Over 90 % respondents from all the socio-economic groups were willing to participate. Multiple household income earners, higher household income, higher number of children, household food security status and higher academic attainment of parents’/caregivers predicted greater willingness to pay. Mean willingness to pay was $4·68 (CAN), and households reporting moderate or severe food insecurity were likely to be willing to pay significantly less for a SFP.

**Conclusion:**

A cost-shared program might be financially sustainable in Canada if community characteristics such as household food insecurity status, economic participation of women and average household size are kept in mind while determining the price of the program.

Across Organisation for Economic Co-operation and Development countries, parents face significant challenges in balancing family and work life which is found to be negatively associated with children’s healthy eating^(1–3)^. Substantial evidence has suggested that the diet quality of Canadian children across the socio-economic spectrum is poor^(4)^ with sugary foods and beverages being a major contributor of daily energy intake^(5)^. Reliance on a energy-dense but not nutrient-dense diet is higher among households with severe food insecurity^(6)^ with negative consequences that are more significant among children living in low-income households^(7,8)^. Numerous chronic disease risk factors first occur in childhood tracking to consequences in adulthood^(9)^. Around two-thirds of Canadian youth have multiple chronic disease risk factors (such as lower levels of physical activity and diet high in sugar), and the distribution is greater among those from lower socio-economic backgrounds^(9)^. The impact is significant for school-going children as inadequate nutritional intake contributes to impaired learning and development^(7)^.

In many countries, public investment has been made in children’s education and in childcare with underlying policy objectives such as improving nutritional intake, child development, increasing fertility rates or reducing gender gaps in employment^(1)^. One such policy intervention has been integrating a school meal program into national legislation, which was done in many affluent countries following the Second World War (WW2). Funding models and other school food policies vary across countries. While in the USA, meals are offered for free or at a reduced cost based on parental income^(10)^, in France meals are subsidised to make them affordable to all students^(11)^. However, unlike its counterpart countries, Canada did not establish a nationally harmonised fully or partially funded school food program (SFP) in the post WW2 period due to a federal policy direction focused on supporting the male breadwinner model^(12)^. As of 2022, Canada remains the only G7 country to not have a national SFP. In the absence of a federally supported school meal program, community-supported child-feeding programs have developed in all the Canadian provinces and territories^(7)^. Some provinces have enacted specific school nutrition policies governing their school food environment while in others school boards are free to develop their own operational standards based on provincial guidelines^(13)^. Most of these policies set nutrient standards governing the type and amount of food that can be offered to children within the school premises and vary substantially in the degree to which they are implemented^(13)^. Consequently, the school food environment in Canada varies widely across regions with most students bringing packed meals from home^(13)^. Given Canada’s unique position of having no current national SFP or policy, and the significant likelihood that one will be established in the years to come^(14)^, analysing whether Canadian caregivers are willing to participate in and to pay for a school food program is a significant step towards devising a policy and implementation plan.

Research has shown the multifaceted benefits of SFP including improving learning outcomes, developing cognitive abilities and improved nutrition^(15)^. Participation in SFP can also reduce socio-economic disparities in fruit and vegetable consumption among adolescents^(16)^. According to recent evidence, SFP are a system-level approach to improve diet-related health outcomes^(17,18)^. SFPs are diverse in their operations, such as taking a universal *v*. targeted approach and full *v*. partial funding^(15)^. Universal SFP refers to a program modality whereby food is accessible to all children irrespective of their family’s financial contribution^(19)^. Research has found that universally offered SFP have higher participation rates compared with eligibility-based programs^(20)^.

A few studies have examined what determines participation in SFP. For example, a study in Vancouver found students’ participation in school-based food and nutrition activities was lower than expected and that it varied by demographic characteristics^(21)^. Lülfs-Baden *et al*. found that by offering healthier meals and communicating the health benefits of the food offered, school meals can be made more attractive to pupils^(22)^. Jensen *et al*. found the price of school lunch, robust planning, school size and feelings of ownership to be key determinants of school lunch viability^(23)^. Another study found stigma, race, age and parental perception influenced participation in school breakfast programs^(20)^. However, most of these studies collected data from students or school administrators, and only a few presented parental or caregiver (hereafter ‘caregiver’) perspectives.

Most research on SFP has focused on the nutritional content of meals and related health impacts. Only a handful have analysed the school food sector from an economic perspective^(22)^. While there are many examples of cost-shared SFP, our review was able to find only a few studies examining caregiver perceptions of sharing the cost of SFP. Filippini *et al*. found among Swiss households that price, household income, satisfaction with the current service, household composition and area of residence were associated with demand for school meal services^(24)^. The study also found that willingness to pay for the services was not dependent upon household income^(24)^. Bere *et al.* found that free school fruit programs were associated with higher intake of fruits and vegetables at school compared with fee-based programs^(25)^.

School food is situated at the nexus between food and education, both of which are considered basic human rights^(26)^. Hence, the idea of charging caregivers for school meals is a potentially contentious topic. This study is not intended to advocate for a cost-shared approach but aims to explore factors that should be kept in mind while considering the various program and implementation modalities. The purpose of our study is to elicit parental willingness to participate and pay for a universally offered SFP as well as factors that determine their decision. We examine caregivers’ demand for a universal SFP by eliciting their willingness to participate and investigate the willingness of caregivers to pay for a portion of such a program. Should caregivers be willing to pay for a portion of the costs of an SFP, a cost-shared model might be politically feasible. In addition, we investigate socio-economic factors that might determine participation in SFP to support future program development. To our knowledge, our study is the first attempt to analyse the Canadian school food sector from an economic and parental willingness perspective.

## Materials and methods

The study was conducted in Saskatoon, Saskatchewan with data collection occurring between October and December 2019. To have representation of neighbourhoods by socio-economic situation, sampling began with a list of Saskatoon Public School Division elementary schools categorised by their location in high, medium or low median-income neighbourhoods using the City of Saskatoon Neighborhood Profiles^(27)^. However, schools situated in these neighbourhoods did not offer the same type of meal programs. Schools could be classified in three groups by their school food situation. These are schools with no meal program, schools having small meal programs (feeding up to 50 % of the pupils) and schools with large meal programs (feeding more than 50 % of the pupils).

Combining this information, four groups were created: (1) low-income neighbourhood schools having large meal programs; (2) low, mid & high-income neighbourhood schools having small meal programs; (3) low- & mid-income neighbourhood schools without any meal program; and (4) high-income neighbourhood schools without any meal program. Finally, three schools were picked at random from each of these groups. Each of the selected school principals was sent the survey link with a request for participation, and the principals were tasked with sending the survey to their students’ caregivers.

### Sampling

The required sample size 390 has been estimated by using the Taro Yamane formula^(28)^. The method is appropriate to use when the only thing known about the population is its size. Two key outcome indicators of our study: the parents/caregivers attitude and demand for a universal cost-shared school meal program has not beem measured in any other study in Canada. In absence of comparable estimates for the two key outcome indicators, the Taro Yamane formula allowed us to estimate required sample size to adequately measure the parents/caregivers attitudes and demand for a universal cost-shared school meal program in Saskatoon.

### Sample size estimation formula

The following formula has been used to calculate the sample size:

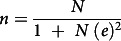




where *n* = corrected sample size, *N* = population size (15 337) and *e* = margin of error (%) (5 %)

### Data collection

A structured questionnaire was used for data collection. The questionnaire was pre-tested with 9 parents representing different socio-economic and cultural backgrounds. Some of the survey questions were modified based on inputs received from pre-testing. Principals of the randomly selected schools were sent the electronic version of the questionnaire and then sent the survey link to the caregivers. Instructions were provided to have only one caregiver per child complete the survey. The questionnaire included sections on demographics, income and household food insecurity, education, ethnicity, attitudes of parents towards SFP and willingness to pay for a cost-shared SFP. 510 parents/caregivers completed the survey out of 965 potential respondents, resulting in a response rate of 52 %.

### Eliciting willingness to participate and pay

A double-bounded dichotomous choice elicitation method was employed to assess the willingness to pay for a potential SFP^(29,30)^. The respondents were given a hypothetical scenario describing an SFP. A basic meal program description was offered that had two key characteristics: (i) it would be offered universally to all kids; and (ii) caregivers/parents could pay for their own child(ren)’s lunches, not pay anything at all or pay extra to help another child whose family could not afford to pay. The program offered would include learning about cooking and gardening while accommodating the various dietary needs of the children.

Using the double bounded dichotomous choice method, the respondents were first asked if they would like to participate in such a school meal program. Respondents who answered affirmatively to this question were considered to be willing to participate in the program. Whether they were willing to pay and how much they were willing to pay were asked only to those who were willing to participate. In this way, we could differentiate between the participants who were willing to participate in the SFP, and participants who were also willing to pay, i.e. willing to join a cost-shared program.

The first bid for the proposed SFP was offered at $4 per meal. While there is minimal data on the cost and the price of SFP offered across Canada, the value was based on the price of school meals offered by the School Lunch Association Canada^(31)^ ^1^. If the parents/caregivers answered ‘yes’ to the first bid, a second higher bid of $8 was offered. If they answered ‘no’ to the initial bid, a second lower bid of $2 was offered. Respondents were also asked to list the maximum amount they would be willing to pay.

### Modeling willingness to pay

Data generated through a contingency valuation method require attention to the censoring or truncation^2^ of the willingness to pay (WTP) value. Often, WTP estimates produce ‘zero’ responses in the form of protest answers. Decisions on whether a participant is willing to join the bidding to pay for the offered product or service and the money they want to spend for that hypothetical product might follow a distinct decision-making process^(32)^. The first decision is whether the participant is willing to participate in the program, i.e. if they are willing to pay at all. This decision may be influenced by ideological or ethical reasons instead of just economic reasons^(32)^. There might be respondents who would like to participate but are not willing to pay for the product or the service. So, the second decision is how much they would be willing to pay if they do want to pay. Such a distinct decision-making process comes with a censored distribution that can be analysed better by a double hurdle (DH) model^(32,33)^. According to the DH model

Y_i_ = Y_i_*; if Y_i_* > 0 and D_i_ > 0

Y_i_ = 0, otherwise

D_i_ = Z_i_θ + μ_i_

where D_i_ is the decision to support the program or not, i.e. willingness to pay, and Yi is the amount willing to pay. Zi is the vector of the independent variables/covariates influencing the decision (Di) and θ is the vector of the parameters^(32)^. In the DH model, Di (willingness to pay) is estimated on the full sample through a logit or Probit model, and Yi (amount willing to pay) is estimated on the sub-sample using a truncated regression model^(32)^.

In a dataset having p covariates 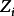 = 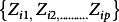 for the *i*th person, where *D_i_*=Willingness to pay (i = 1 = Willingness to pay, i = 0 = not willing to pay), in the first stage of the DH model, we model the odds of paying for the school meal program on the covariates via a logistic regression as

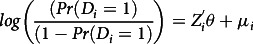




where 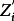 is the vector of the covariates, and 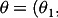 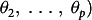 is the vector of parameters.

The final empirical model for willingness to pay is

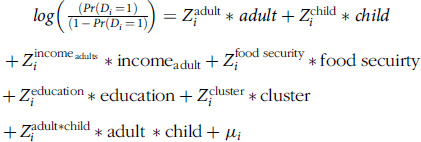




The same set of covariates are used in the truncation regression (the second part of the DH model), which we present as

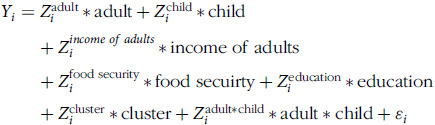




As our data is truncated from above at $8 and truncated from below at $2, we will observe *Y_i_* and 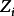 between 2 and 8 CAD.

### Data analysis

*χ* ^2^ analyses were performed to examine the distribution of the independent variables on both willingness to participate and pay. Both simple and multivariable logistic regression models were employed to determine factors associated with participants’ willingness to participate. We employed a double hurdle model (which include a logit and a truncated regression model) to estimate participants’ WTP. Crude estimates of association were calculated by fitting separate simple logistic regression models between the dependent variables and a set of independent variables. The multivariable logistic regression models were fitted with the independent variables that were deemed significant (*P* < 0·20) based on simple logistic regression analysis. For both willingness to participate and pay, all variables that had *P* value < 0·05 were retained in the final logit model. Finally, a truncated regression model was employed to determine the amount of willingness to pay. Table 1 lists all the independent variables we tested to describe participants’ willingness to participate and pay for a universal and cost-shared school meal program. The mean of WTP was derived following Lopez–Feldman under the contingency valuation method using STATA 17^(34)^.

**Table 1:** List of covariates used to understand respondent’s willingness to participate and pay in a universal and cost-shared school meal program

Variables	Explanation	Measurement	Expectation	Source
Child	Number of children under the 18	0 = 1 child 1 = 2 child 2 = 3 or more child	Higher number of children under 18 will entail higher willingness to participate but lower willingness to pay	Drafted by the research team
Adult	Number of adults in the household	0 = 1 adult 1 = 2 adult 2 = 3 or more adults	Higher number of adults in the family will have lower willingness to participate and lower willingness to pay	Drafted by the research team
Income_adults	Total number of adults bringing income	0 = 1 adult 1 = 2 or more adult	Higher number of adults bringing income will have higher willingness to participate and higher willingness to pay	Drafted by the research team
Education	Measures the highest level of education attained by the respondent	0 = less than secondary school, 1 = Completed secondary school, 2 = attainment of any post-secondary education	Higher level of education will entail a higher willingness to participate and higher willingness to pay	Drafted by the research team
Ethnicity	Measures self-identification of ethnic heritage of respondents	0 = White, 1 = Indigineous, 2 = Visible minorities	Respondents with visible minority status will have higher willingness to participate and pay	Canadian Community Health Survey (CCHS)^(35)^
Sfp	Measure the current status of school food offering in the schools	0 = No SFP, 1 = small SFP (serving < 50 % children), 2 = large SFP (serving more than 50 % children)	Parents from schools with large SFP coverage will have higher willingness to participate and pay	Developed based on our conversation with the school board and practitioners.
Food security	Measures household food security status	0 = Food secure, 1 = moderate food insecurity, 2 = severe food insecurity.	People with food insecurity will have higher willingness to participate but lower willingness to pay	Household Food Security Survey Module (HFSSM) from the Canadian Community Health Survey (CCHS)^(35)^
Cluster	Classifies the schools by the socio-income status of the neighbourhood and situation of school food program	0 = Low-income neighbourhood schools with large SFP, 1 = low-, mid- and high-income neighbourhood schools with small SFP, 2 = low- and mid-income neighbourhood schools with no SFP, 3 = high-income neighbourhood schools with no SFP	Parents from no school meal program will lean more towards having a school meal program	Developed based on our conversation with the school board and practitioners.
Adult*child	Measures if there is any interaction between number of adults and number of child members in the household.			

## Results

Table 2 summarises the descriptive statistics of the study. 86 % of the study participants were women. Average household size of the study sample was 4. The average number of children under 18 was a little over 2 and the average number of adults bringing income was 1·72. Less than one-third of the survey respondents were Indigenous and other visible minorities. Over 80 % of parents had post-secondary education and over half of them were working full-time. Around one-fifth of the participants were unemployed, and around two-thirds of participants reported earning less than 9000 CAD as total monthly household income (less than 108 000 yearly). Around half of respondents had children who attended schools located in low-and mid-income neighbourhoods and around 7 % of respondents reported severe food insecurity on the household scale. Over 60 % of caregivers reported sending children to schools that did not have a school meal program.

**Table 2:** Frequency and mean/percentage of survey population by household characteristics, parental attributes and socio-economic factors

Variable	Survey sample	
	Frequency (*n*)	Mean
Household characteristics		
Average household size	474	4·09
Average number of children under 18	498	2·13
Average number of children attending school	504	1·62
Average number of adults in household	498	1·98
Average number of adults bringing income	503	1·72
Parental characteristics		
Average age, years	478	39·19
	*n*	%
Sex		
Female	434	86
Male	71	14
Ethnic identity		
White	370	74
Indigenous	51	10
Other visible minorities	81	16
Education		
Less than secondary school	47	9
Secondary school complete	42	8
Post-secondary	416	82
Occupation		
Unemployed^3^	125	25
Working full time	293	58
Working part-time	89	18
Socio-economic characteristics		
Total household income (monthly)		
Low income (less than $4000)	140	33
Mid income ($4000–$9000)	144	34
High income (More than $9000)	136	32
Household food insecurity		
Moderate	85	17
Severe	38	7
Income status of school’s neighbourhood		
Low	140	32
Medium	82	19
High	210	49
Schools by neighbourhood income and status of meal program offered		
Low-income neighbourhood schools with large SFP	19	4
Low-, mid- and high-income neighbourhood schools with small SFP	137	32
Low- and mid-income neighbourhood schools with no SFP	129	30
High income neighbourhood schools with no SFP	147	34
Current School Food Program		
Child participating in school meal program (no/yes)	79	15
Coverage of current SFP		
No SFP	276	64
Small SFP (serving less than 50 % children)	137	32
Large SFP (serving more than 50 % children)	19	4

**Fig. 1:**
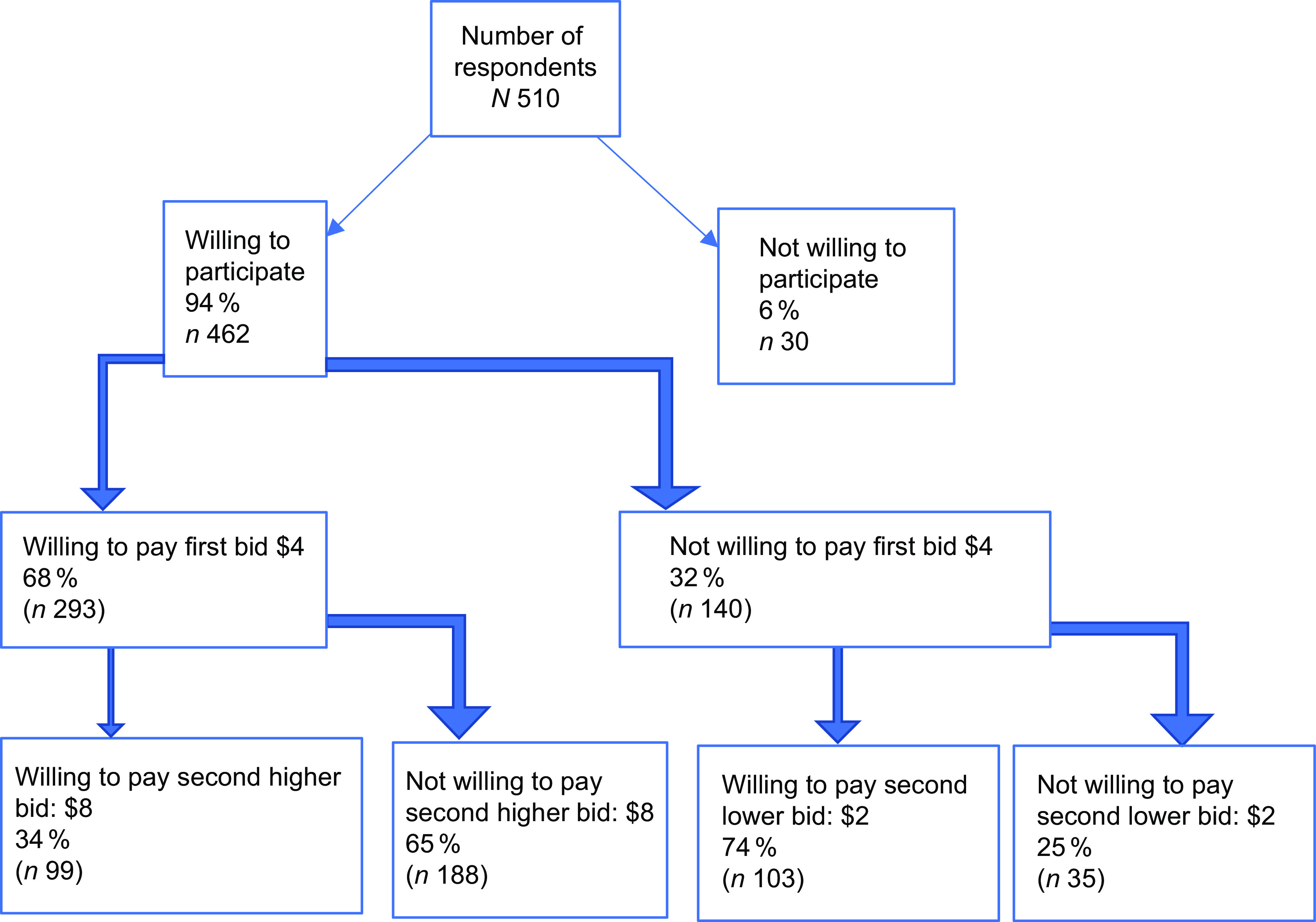
Summary statistics for the willingness to pay module

Figure 1 shows the summary statistics for the willingness to participate and pay module questions. Of the 510 participants, 462 (94 %) agreed to participate in the proposed SFP. Of the respondents willing to participate, 68 % agreed to pay the first bid of $4 per child per meal, while 32 % were unwilling to pay the first bid amount. When the first bid was increased to $8 per child per meal, 34 % (*n* 99) of those who agreed to pay the first bid were still willing to pay the second bid and 65 % (*n* 188) were unwilling to pay. Of the respondents who were not willing to pay the first bid of $4, a lower bid of $2 per child per meal was offered. 74 % (*n* 103) of the participants that were unwilling to pay first were now willing to pay for the program. Mean willingness to pay was $4·68/d per child (figure not shown).

Table 3 shows the proportion of respondents willing to participate and pay in the proposed school SFP within the categories of various socio-economic factors with their associated *χ* ^2^ analysis. The *χ* ^2^ analysis showed that more than 90 % of parents in almost all the subgroups were willing to participate in the proposed SFP. People with higher education and less household income were more willing to participate. All participants with severe food insecurity (in household scale) were willing to participate and participants from low-income neighbourhoods were also more willing to participate; however, differences within these categories were not statistically significant.

**Table 3:** Frequency distribution and *χ* ^2^ analysis of the willingness to participate and willingness to pay by household characteristics, parental attributes and socio-economic factors

Variables	Willingness to participate %	*n*/*N*	*χ* ^2^	*P* value	Willingness to pay %	*n*/*N*	*χ* ^2^	*P* value
Household size								
2–3 members	97	102/105	4·99	0·08	74	83/112	3·52	0·172
4–5 member	92	305/331			80	271/336		
More than 5 members	100	23/23			69	18/26		
Number of children under 18								
1 child	96	94/98			77	81/105		
2 children	93	238/256			80	209/260		
3 or more children	94	119/127	1·06	0·59	73	97/133	2·84	0·241
Number of adults in the household								
1 adult	98	55/56			70	42/60		
2 adults	93	361/386			81	321/396		
3 or more adults	92	35/38	2·15	0·34	59	25/42	12·71	0·002
Number of adults bringing income								
1 adult	95	133/140			66	97/148		
2 or more	93	323/346	0·47	0·49	83	296/355	19·45	<0·001
Ethnic identity								
White	93	332/358			81	300/370		
Indigenous	96	47/49			71	36/51		
Other visible minorities	99	76/77	4·35	0·11	68	55/81	8·45	0·015
Sex of parents								
Female	95	396/419			78	339/434		
Male	93	63/68	0·38	0·54	77	55/71	0·01	0·903
Parental characteristics								
Education								
Less than secondary	86	38/44			70	33/47		
Secondary school graduation	93	37/40			62	26/42		
Post-secondary	95	382/403	5·01	0·08	80	334/416	9·20	0·01
Occupation								
Unemployed	93	109/117			67	84/125		
Working full time	94	266/283			82	239/293		
Working part-time	94	84/89	0·28	0·96	80	71/89	11·31	0·01
Socio-economic characteristics								
Total household income (monthly)								
Low income (< $4000)	96	128/134			69	96/140		
Mid income ($4000–$9000)	94	132/141			83	120/144		
High income (More than $9000)	93	124/134	1·06	0·586	89	121/136	19·42	<0·001
Food insecurity (household scale)								
Food secure	93	350/375			83	320/387		
Moderate food insecurity	94	77/82			64	54/85		
Severe food insecurity	100	35/35	2·48	0·289	61	23/38	21·96	<0·001
Neighbourhood income status								
Low	96	128/133			74	103/140		
Medium	95	73/77			76	62/82		
High	92	190/206	2·41	0·30	85	178/210	7·32	0·026
Coverage of school meal program								
No meal program	96	255/266			83	228/276		
Small meal program	90	120/133			78	107/137		
Large meal program	94	16/17	4·99	0·08	42	8/19	18·03	<0·001

Table 3 also presents the proportion of participants who were willing to pay for the proposed SFP. The *χ* ^2^ analysis showed that households’ ethnic identity, number of adult household members, number of employed adult members, caregivers’ educational status, total household income, household food insecurity status and the coverage of current SFP (no *v*. small *v*. large meal programs) were all statistically significant. Of those who attained post-secondary education and who were working full-time, around 80 % were willing to pay. Participants’ WTP declined as the number of household members increased. Participants’ WTP was also higher in those households where two or more adults were bringing in income. People from food secure households were more willing to pay compared with households with moderate or severe food insecurity. Parents from high-income neighbourhoods were also more willing to pay compared with parents from low- or medium-income neighbourhoods. A high income of more than $9000 (monthly) accompanied a greater WTP. As most of our study participants were female, we did not find a difference based on the sex of the parent/caregiver of the child.

We performed a multivariable logistic regression to explore the factors associated with participants’ willingness to participate in the proposed SFP (Table 4). Factors that were statistically significant (*P* < 0·20) in the simple logistic regression models were kept in the multivariable model. Only participants’ educational attainment and coverage of the school meal program retained statistical significance in the final model indicating a caregiver’s education and coverage of current SFP significantly predicted their willingness to participate in school meal programs. Results indicate that parents whose children went to a school with small to medium SFP were less likely to want to participate in the proposed SFP compared with the participants whose children went to a school without an SFP. Participants with secondary education or more were more likely to want to participate in an SFP compared with parents with less than high school education.

**Table 4:** Factors associated with willingness to participate in a cost-shared school meal program in Saskatoon, Canada (estimates of odds ratios and 95 % confidence intervals (CI))

Variables	OR	95 % CI	*P* value
Ethnic identities			
White	Ref		
Indigenous	1·40	0·30, 6·51	0·666
Other visible minorities	3·64	0·47, 28·21	0·215
Education			
Less than secondary	Ref		
Secondary school graduation	1·32	0·27, 6·33	0·725
Post-secondary	2·60	0·87, 7·76	0·086
Coverage of school food program			
No meal program	Ref		
Small	0·38	0·16, 0·92	0·032
Large	0·70	0·07, 6·47	0·758

Table 5 shows the findings of the double hurdle model to explore the factors associated with participants’ WTP for the proposed SFP. In another words, it shows participants’ willingness to join a cost-shared SFP and factors associated with it. The multivariable logit model contained all the statistically significant factors of the individual logistic regression models. The number of household members, number of children in the household, number of adult members bringing income, household food insecurity and education status of parents/caregivers were significantly associated with WTP for an SFP.

**Table 5:** Factors associated with willingness to pay in a cost-shared school meal program in Saskatoon, Canada (estimates of odds ratios and coefficients) based on double hurdle model

Variables	Double hurdle model on willingness to pay			
	Willingness to pay		Amount willing to pay	
	Logit model (WTP = 0 is base)		Truncated regression model (WTP > 0 is base)	
	OR	*P* value	Coef.	*P* value
Number of adults in the household				
1	Ref		Ref	
2	0·57	0·436	−0·46	0·555
3 or more	0·12	0·025	−1·34	0·301
Number of children under 18				
1	Ref		Ref	
2	0·22	0·050	−0·71	0·502
3 or more	4·78	0·245	−2·10	0·064
Number of adults bringing income				
1	Ref		Ref	
2 or more	2·39	0·006	1·18	0·002
Household food insecurity				
Food secure	Ref		Ref	
Moderate food insecurity	0·52	0·065	−1·90	<0·001
Severe food insecurity	0·58	0·286	−1·55	0·039
Education				
Less than secondary school	Ref		Ref	
Secondary school graduation	2·90	0·294	2·56	0·294
Post-secondary	5·22	0·099	2·58	0·286
Cluster				
Low-income neighbourhood schools with large SFP	Ref		Ref	
Low-, mid- and high-income neighbourhood schools with small SFP	3·30	0·057	0·33	0·767
Low- and mid-income neighbourhood schools without SFP	2·98	0·086	1·04	0·362
High income neighbourhood schools without SFP	4·05	0·034	0·83	0·462
Interaction terms				
Number of adult and child household members	0·062			

The results indicated that households with two or more adults present were less willing to share the cost of the proposed SFP. However, households with more than one adult income earner were two times more likely to be willing to pay for the SFP compared with households comprising one income earner. Households with two children were less likely to be willing to pay for an SFP, whereas households with three or more children were four times more willing to pay for an SFP compared with households with one child only. Households with moderate or severe levels of food insecurity were less willing to pay for SFP compared with food secure households. Parents/caregivers who completed post-secondary education were five times more likely to be willing to pay for the proposed SFP compared with parents with no post-secondary education.

Parents/caregivers were more likely to be willing to pay for an SFP if their children did not go to a school offering a food program already, irrespective of the socio-economic status of the neighbourhood where their schools were located. In addition, we found the number of adult household members interacted significantly with the number of children of the household to determine the household’s WTP.

Table 5 also shows the results of our truncated regression model depicting the amount households are willing to pay. Households with more than one income earner are likely to pay a dollar more compared with households with a single income earner. Households with moderate or severe food insecurity are likely to be willing to pay significantly less compared with food secure households. Households with higher education are willing to pay more although this is not statistically significant.

## Discussion

Our study found a significant demand for a universally offered SFP among caregivers in Saskatoon, Canada. Over 90 % respondents were willing to participate in the proposed SFP. Willingness to pay declined as price increased, and factors that predicted respondents’ willingness to share the cost of the food program were number of income earners in the household, number of children in the household, parental education and household food insecurity status. However, it should be noted that these findings are conditional on the specific model offered – a universal SFP that would consider pupils’ dietary needs, integrating food and cooking related knowledge into classroom learnings.

Median household income in Saskatoon is around $67, 000/year^(36)^. While around half of our survey participants (49 %) came from high-income neighbourhoods, almost one-fourth (24 %) of them reported being food insecure (moderate and severe) signifying hidden food insecurity even in affluent sections of society. The proportion of people willing to participate and pay declined with the severity of household food insecurity. Severely food-insecure households were significantly less willing to pay for an SFP. Interestingly, in our study, participants whose children were already enrolled in a school offering a food program were less willing to participate in the proposed SFP. This could mean that apart from socio-economic factors that might influence the decision to participate, concern about the quality or other aspects of the meals offered and fear of stigma associated with participation in SFP might be a key reason behind the participation decision. For example, Forrestal *et al*. found that participation in SFP was higher in schools that offered universal free school meals because it eliminated the risk of social stigma due to school meal participation^(37)^. This, in particular, has significant policy implications.

In our study, WTP for an SFP was significantly higher among dual-earner households and among households with more than three children. While around 150 of our study respondents had three or more children, the majority had two children, and WTP was lower for respondents with two children. The fertility rate in Canada has been declining significantly over the last few decades, and the current total fertility rate is around 1·61. Therefore, there is a possibility that the proportion of people willing to pay would be a little lower than what it is now, if inferred to the broader population of Canada. Nevertheless, evidence shows that parents today face significant challenges to cope with demands on time irrespective of the number of children they might have. Standing in the nexus of balancing between socio-cultural norms, changing lifestyles, financial situation and time, parents might rely on food provisioning choices leading to bad nutritional outcomes such as relying on highly processed convenience foods when preparing lunches for their children^(2)^. SFP can play a crucial role in this context by offering nutritious foods made from basic ingredients.

There need to be more studies examining caregivers’ demands and attitudes towards school meal programs, the absence of which limits our capacity to compare our study findings with others. However, our study’s findings closely relate to other studies examining parents’ WTP for various childcare programs. For example, parents’ WTP has been found to be usually higher for preventive programs such as childhood obesity prevention programs, and WTP was associated with parental income^(38)^. Catma *et al.* found that parents WTP for COVID-19 vaccines was higher and it increased with the number of children in the household^(39)^. As found in other studies, education was a significant predictor of both willingness to participate and pay. Respondents with higher academic attainments were more willing to participate and pay for a school meal program.

Our findings are significant for multiple reasons and should be discussed in their entirety. The clear distinction between the proportion of respondents willing to participate and willing to pay for a SFP has significant implications. That over 95 % of the participants wanted to participate in a universal SFP which drops below 70 % for a cost-shared scheme implies that even though there is overwhelming demand for a universal SFP, government should be careful in choosing a cost-shared modality. Support for a cost-shared scheme were seen mostly among dual-earner households, households with more children and parents with higher education. It was significantly lower among people with higher food insecurity. Cost of the program, fear of stigma^(40)^, meal quality as well as ethical imperatives might play a role in parents’ views on cost sharing. However, our findings also point to the role SFP can play in helping parents maintain work-life balance while ensuring healthy meals for their children. Hence, while establishing an SFP should be a policy priority, policymakers and stakeholders should devise a plan that minimises the risks associated with a cost-shared approach. Attention should be paid in provinces with higher levels of food insecurity so that meals are affordable to the majority of families. A universally offered programme that considers children’s dietary needs, with an implementation modality that keeps the status of caregivers’ contributions confidential is crucial. The establishment of a specific national policy for an SFP in Canada needs to integrate multiple sectors such as health, education, agriculture and multiple levels of government such as federal, provincial and municipal. Although most countries offer school meal programs, only a handful offer them universally. Brazil, for example, is one of the few countries offering school meal programs universally to ensure food and nutrition security^(41)^. The SFP in Japan covers more than 90 % of elementary schools and aims at imparting food and nutrition related knowledge to pupils from an early age^(42)^. School meal programs in the USA have a strong legislative structure and fiscal base, while in Italy, parent-led associations serving organic food are slowly gaining acceptance. In most countries, parents are involved in these programmes, which is crucial to ensure children’s participation. Therefore, while a thorough review of school food policy and implementation modalities is critical, understanding what parents/caregivers of children want is also crucial to developing Canada’s school food policy and practices.

Some potential limitations might apply to our study. It was conducted through an online platform that usually entails a low response rate. The electronic link to the survey was sent to the selected school principals, who emailed the parents with the survey link. Completing the online survey was contingent upon a few things, such as parents having an active email address, some technical knowledge to access and complete the survey, and access to a device and internet connection to complete the survey. To minimise the low response rate due to these reasons, we offered to have our research assistants visit schools during a parent–teacher event with electronic devices to help the parents fill out the survey. However, most schools declined the offer as our presence would have the risk of influencing the survey responses. Some parents opened the survey link but did not submit the responses. However, as we sent the survey link to schools by categorising them by their socio-economic status and school meal situation, the non-response rate is unlikely to bias the survey estimates. In addition, some variables, such as caregivers’ ethical standpoint (whether school meals should be cost-shared) or political interests, may also impact caregivers’ willingness to join and pay. However, these are difficult to capture, and we decided not to do so in our survey. We were not able to collect information on the parents who did not complete the survey and so we do not know the distribution of non-response across the clusters of caregivers.

## Conclusion

This is an exciting time for the school food sector in Canada as school food advocates, parents, researchers and policymakers are calling for the initiation of a national SFP and governments are beginning to respond^(14)^. In the March 2019 federal budget, the Government of Canada announced a commitment towards a school meal program^(14)^, and soon after the provincial government in Quebec allocated $11 million to extend eligibility of SFP^(43)^. Other provinces have also begun to make similar commitments. In considering the factors most significantly associated with willingness to participate and pay for children’s caregivers, policymakers can design a program that will meet the needs of the vast majority of families in Canada.

## Footnotes

1 School Lunch association is a registered charity that offers school lunch in around 36 schools across Canada. Even though the organization offers a ‘pay what you can’ model, the suggested price per meal is $ 3·75^(31)^.

2 Following DBDC method, WTP data is censored as it could not go above or below a certain threshold.

3 Unemployment includes people without any paid job at the time of the survey, and it also includes people who indicated that they were looking for work, retired, staying home to look after children.

## References

[1] The Organization for Economic Cooperation and Development (2005) Babies and Bosses: Reconciling Work and Family Life (Vol 4): Canada, Finland, Sweden and the United Kingdom. Paris: OECD Observer.

[2] Slater J , Sevenhuysen G , Edginton B et al. (2012) “Trying to make it all come together”: structuration and employed mothers’ experience of family food provisioning in Canada. Health Promot Int 27, 405–415. 21693474 10.1093/heapro/dar037

[3] Bauer KW , Hearst MO , Escoto K et al. (2012) Parental employment and work-family stress: associations with family food environments. Soc Sci Med 75, 496–504. 22591825 10.1016/j.socscimed.2012.03.026 PMC3586574

[4] Ahmadi N , Black JL , Velazquez CE et al. (2015) Associations between socio-economic status and school-day dietary intake in a sample of grade 5–8 students in Vancouver, Canada. Public Health Nutr 18, 764–773. 25098190 10.1017/S1368980014001499 PMC10271573

[5] Hack S , Jessri M & L’Abbé MR (2021) Nutritional quality of the food choices of Canadian children. BMC Nutr 7, 16. 34049592 10.1186/s40795-021-00422-6 PMC8164219

[6] Hutchinson J & Tarasuk V (2022) The relationship between diet quality and the severity of household food insecurity in Canada. Public Health Nutr 25, 13–26. 34551845 10.1017/S1368980021004031 PMC9991759

[7] Raine K , McIntyre L & Dayle JB (2003) The failure of charitable school- and community-based nutrition programmes to feed hungry children. Crit Public Health 13, 155–169.

[8] St John M , Durant M , Campagna PD et al. (2008) Overweight Nova Scotia children and youth: the roles of household income and adherence to Canada’s Food Guide to Healthy Eating. Can J Public Health 99, 301–306. 18767276 10.1007/BF03403760 PMC6976246

[9] Alamian A & Paradis G (2009) Clustering of chronic disease behavioral risk factors in Canadian children and adolescents. Prev Med 48, 493–499. 19254742 10.1016/j.ypmed.2009.02.015

[10] US Department of Agriculture (2022) The National School Lunch Program. https://www.fns.usda.gov/nslp (accessed April 2022).

[11] Moffat T & Thrasher D (2016) School meal programs and their potential to operate as school-based obesity prevention and nutrition interventions: case studies from France and Japan. Crit Public Health 26, 133–146.

[12] Carbone S , Power E & Holland MR (2020) Canada’s missed opportunity to implement publicly funded school meal programs in the 1940s. Crit Public Health 30, 191–203.

[13] Phorson J (2015) Policies and Guidelines Shaping the School Food Environment: A Review of the Literature Prepared for the Nutrition Resource Centre Introduction. Ontario: Nutrition and Resource Centre.

[14] Government of Canada (2019) Budget 2019: Chapter 4 Delivering Real Change. https://www.budget.canada.ca/2019/docs/plan/chap-04-en.html (accessed March 2022).

[15] Bundy D , Burbano C , Grosh M et al. (2009) Rethinking School Feeding: Social Safety Nets, Child Development, and the Education Sector. Directions in Development – Human Development. Washington, DC: The World Bank.

[16] Longacre MR , Drake KM , Titus LJ et al. (2014) School food reduces household income disparities in adolescents’ frequency of fruit and vegetable intake. Prev Med 69, 202–207. 25456807 10.1016/j.ypmed.2014.10.008 PMC4312181

[17] Hernandez K , Engler-Stringer R , Kirk S et al. (2018) The case for a Canadian national school food program. Can Food Stud 5, 208–229.

[18] Lee BY , Bartsch SM , Mui Y et al. (2017) A systems approach to obesity. Nutr Rev 75, Suppl. 1, 94–106. 28049754 10.1093/nutrit/nuw049 PMC5207008

[19] Food Secure Canada (2021) What is Meant by a “Universal Student Nutrition Program”? Say Yes! to Good Healthy Food in Schools. https://foodsecurecanada.org/resources-news/resources-research/universal-snp-say-yes (accessed April 2022).

[20] Lopez-Neyman S & Warren C (2016) Barriers and advantages to student participation in the school breakfast program based on the social ecological model: a review of the literature. J Child Nutr Manag 40, 1–13.

[21] Stephens TA , Black JL , Chapman GE et al. (2016) Participation in school food and nutrition activities among grade 6–8 students in Vancouver. Can J Diet Pract Res 77, 148–153. 27182726 10.3148/cjdpr-2016-003

[22] Lülfs-Baden F , Rojas-Mendez J & Spiller A (2008) Young consumers’ evaluation of school meals. J Int Food Agribus Mark 20, 25–57.

[23] Dejgård Jensen J , Smed S , Raun Mørkbak M et al. (2013) Economic viability of new launched school lunch programmes. Br Food J 115, 1038–1053.

[24] Filippini M , Masiero G & Medici D (2014) The demand for school meal services by Swiss households. Ann Public Coop Econ 85, 475–495.

[25] Bere E , Veierød MB & Klepp K-I (2005) The Norwegian school fruit programme: evaluating paid *v*. no-cost subscriptions. Prev Med 41, 463–470. 15917042 10.1016/j.ypmed.2004.11.024

[26] United Nations (1948) United Nations General Assembly Resolution 217 A: Universal Declaration of Human Rights. https://www.un.org/en/development/desa/population/migration/generalassembly/docs/globalcompact/A_RES_217(III).pdf (accessed April 2022).

[27] Planning and Development City of Saskatoon (2021) City of Saskatoon Neighbourhood Profile. https://www.saskatoon.ca/sites/default/files/documents/community-services/planning-development/research/neighbourhood-profiles/neighbourhood_profiles_2019.pdf (accessed April 2022).

[28] Yamane T (1967) Statistics: An Introductory Analysis, 2nd ed. New York: Harper and Row.

[29] Hanemann M , Loomis J & Kanninen B (1991) Statistical efficiency of double-bounded dichotomous choice contingent valuation. Am J Agric Econ 73, 1255–1263.

[30] Cerda AA & García LY (2021) Willingness to pay for a COVID-19 vaccine. Appl Health Econ Health Policy 19, 343–351. 33619688 10.1007/s40258-021-00644-6 PMC7899739

[31] The School Lunch Association (2019) School Lunch. https://schoollunch.ca/our-story/ (accessed April 2022).

[32] Guo Z & McDonnell S (2013) Curb parking pricing for local residents: an exploration in New York City based on willingness to pay. Transp Policy 30, 186–198.

[33] Cragg JG (1971) Some statistical models for limited dependent variables with application to the demand for durable goods. Econometrica 39, 829–844.

[34] StataCorp. (2021) Stata Statistical Software: Release 17. College Station, TX: StataCorp. LLC.

[35] Statistics Canada (2017) Reference Guide to Understanding and Using the Data: 2015 Canadian Community Health Survey – Nutrition. https://www.canada.ca/en/health-canada/services/food-nutrition/food-nutrition-surveillance/health-nutrition-surveys/canadian-community-health-survey-cchs/reference-guide-understanding-using-data-2015.html (accessed April 2022).

[36] Statistics Canada (2022) Table 2: Median After-Tax Income, Canada and Provinces, 2016 to 2020. https://www150.statcan.gc.ca/n1/daily-quotidien/220323/t002a-eng.htm (accessed April 2022).

[37] Forrestal S , Potamites E , Guthrie J et al. (2021) Associations among food security, school meal participation, and students’ diet quality in the first school nutrition and meal cost study. Nutrients 13, 307. 33499016 10.3390/nu13020307 PMC7912040

[38] Kesztyüs D , Lauer R , Schreiber AC et al. (2014) Parents’ willingness to pay for the prevention of childhood overweight and obesity. Health Econ Rev 4, 20. 26208923 10.1186/s13561-014-0020-8 PMC4883987

[39] Catma S & Varol S (2021) Willingness to pay for a hypothetical COVID-19 vaccine in the United States: a contingent valuation approach. Vaccines 9, 318. 33915680 10.3390/vaccines9040318 PMC8065984

[40] Downey M (2020) No Such Thing as a Stigma-Free Lunch. The Regulatory Review. https://www.theregreview.org/2020/10/21/downey-no-such-thing-stigma-free-lunch/ (accessed April 2022).

[41] Sidaner E , Balaban D & Burlandy L (2013) The Brazilian school feeding programme: an example of an integrated programme in support of food and nutrition security. Public Health Nutr 16, 989–94. 23218237 10.1017/S1368980012005101 PMC10271776

[42] Coalition for Healthy School Food (2022) Japan’s School Food Program Webinar. https://www.youtube.com/watch?v=6VyHE4vO12g (accessed April 2022).

[43] Canadienne P (2020) Quebec Extends Eligibility for school Meal Program, Increases Budget by 60 %. Montreal Gazette. https://montrealgazette.com/news/local-news/quebec-extends-eligibility-for-school-meal-program-increases-budget-by-60#:∼:text=Local%20News-,Quebec%20extends%20eligibility%20for%20school%20meal%20program%2C%20increases%20budget%20by,those%20funds%20will%20be%20revised (accessed April 2022).

